# Synergistic Effects of Resveratrol and Pyrimethanil against *Botrytis cinerea* on Grape

**DOI:** 10.3390/molecules23061455

**Published:** 2018-06-15

**Authors:** Dandan Xu, Ge Yu, Pinggen Xi, Xiangyu Kong, Qi Wang, Lingwang Gao, Zide Jiang

**Affiliations:** 1 Department of Plant Pathology/Guangdong Province Key Laboratory of Microbial Signals and Disease Control, South China Agricultural University, Guangzhou 510642, China; happyxudandan@126.com (D.X.); yg8335266@163.com (G.Y.); xpg@scau.edu.cn (P.X.); kong19941124@163.com (X.K.); 2 College of Plant Protection, China Agricultural University, Beijing 100193, China; wangqi@cau.edu.cn

**Keywords:** adjuvant, antifungal activity, drug combination, gray mold, resistant isolate

## Abstract

*Botrytis cinerea* is the pathogen of gray mold disease affecting a wide range of plant hosts, with consequential economic losses worldwide. The increased frequency of fungicide resistance of the pathogen challenges its disease management, and thus the development of alternative control strategies are urgently required. In this study, we showed excellent synergistic interactions between resveratrol and pyrimethanil. Significant synergistic values were recorded by the two-drug combination on the suppression of mycelial growth and conidia germination of *B. cinerea*. The combination of resveratrol and pyrimethanil caused malformation of mycelia. Moreover, the inoculation assay was conducted on table grape and consistent synergistic suppression of the two-drug combination was found in vivo. Our findings first revealed that the combination of resveratrol and pyrimethanil has synergistic effects against resistant *B. cinerea* and support the potential use of resveratrol as a promising adjuvant on the control of gray mold.

## 1. Introduction

*Botrytis cinerea* is an airborne phytopathogenic fungus responsible for invasive gray mold causing significant economic losses during harvesting and subsequent handling, storage, marketing, and after consumer purchase [1,2]. Control of gray mold based on chemical fungicide application in the field is mainly achieved by botryticides belong to benzimidazoles, anilinopyrimidines and dicarboximides [3]. The anilinopyrimidine fungicides such as pyrimethanil, cyprodinil and mepanipyrim have been launched for the control of various ascomycetes pathogens, especially the gray mold on fruits, vegetables and ornamentals [4,5]. Anilinopyrimidines are synthetic drugs and although effective, resistant isolates have been frequently reported due to the increasing amounts of applied fungicides [6,7]. Moreover, *B. cinerea* is one of the high frequencies of drug-resistant pathogens due to its high genetic variability, short life cycle, and prolific reproduction [8]. Furthermore, excessive fungicide application causes various side effects on human health and environment [9], and therefore it is of great importance to search for novel active ingredients with potent antifungal activity via targeted modes of action to mitigate against resistance development.

Resveratrol, a natural phenolic compound is widespread in higher plant and a vital target in drug discovery due to its role in plant defense against different stress conditions such as pathogen invasion [10,11], UV radiation [12,13,14], temperature variation [15] and severe drought [16]. Also, resveratrol affects a broad range of biological processes due to its broad-spectrum antimicrobial [17,18,19], antiviral activities [20] as well as growth modulatory effects on various organisms [21]. Moreover, resveratrol displays significant antifungal effects toward several plant pathogens [22,23,24], and our previous study indicated the excellent efficacy of resveratrol on the control of gray mold of table grape [25]. Various pharmacological and biological properties of resveratrol have made it an attractive candidate for the plant disease control.

Combination therapy of two or more antibiotics has emerged as an attempt to prevent or delay the emergence of resistance, enhance the activity of antibiotics and reduce the application amounts of fungicides [26,27]. Plant phenolics, as natural secondary metabolites, have been reported to enhance the activity of classical antibiotics and represent promising adjuvants of antibacterial and antifungal drugs [28,29,30]. To our knowledge, however, no studies have been conducted on the synergistic effects of phenolic compounds in combination with fungicides against *B. cinerea*.

In this study, the antifungal activity of selected phenolic resveratrol alone and in combination with conventional fungicide pyrimethanil against *B. cinerea* was evaluated by microdilution method. Additionally, their synergistic effects on mycelial growth and conidia germination of *B. cinerea* in vitro, and the suppression of table grape gray mold disease were also explored. 

## 2. Results

### 2.1. Sensitivity of B. cinerea Isolates to Pyrimethanil and Cyprodinil

The EC_50_ values for inhibition of mycelial growth of *B. cinerea* isolates were used to determine the sensitivity of tested isolates to anilinopyrimidine fungicides pyrimethanil and cyprodinil. Six *B. cinerea* isolates, GBW, TGM, SGB, GMR, BGM and BRB, named according to the location and cultivar of infected grape berries, were tested. Among the tested isolates, four isolates (GBW, TGM, SGB and GMR) were classified in categories with EC_50_ values ranges from 4.5 to 45 mg/L and exhibited moderate resistance to pyrimethanil, two isolates (BGM and BRB) exhibited high resistance to pyrimethanil with the EC_50_ values >70 mg/L (Table 1). For cyprodinil, the GMR isolate was sensitive with an EC_50_ value of 1.3 mg/L. Three isolates including TGM, SGB and BRB had an EC_50_ value ranged from 10 to 20 mg/L and were considered as moderately resistant isolates, and two isolates GBW and BGM were resistant to cyprodinil (Table 1). In this study, only one isolate was sensitive to cyprodinil, the other five tested isolates displayed moderate to high resistance to pyrimethanil, and moderate to complete resistance to cyprodinil. Moreover, all tested isolates had the EC_50_ values hundred times higher than 0.09 mg/L pyrimethanil, the baseline sensitivity level reported by Ji et al. [31]. Therefore, pyrimethanil was selected in this study to search for suitable natural compounds as synergist on delaying/blocking drug-resistance development and reducing application amount of fungicides.

### 2.2. Determination of MICs and Interaction Analysis

The MICs of pyrimethanil alone or in combination with resveratrol against *B. cinerea* are shown in Table 2. The MICs of pyrimethanil alone against moderately resistant isolates (GBW, TGM, SGB and GMR) and highly resistant isolates (BGM and BRB) were 0.625–1.25 mg/L and 2.5 mg/L, respectively. The MICs of pyrimethanil in combination with resveratrol were reduced to 0.07813–0.3125 mg/L. Similarly, the MICs of resveratrol alone were 5–80 mg/L and were reduced to 1.25–5 mg/L when combined with pyrimethanil. The in vitro antifungal activity assay showed that the MICs of pyrimethanil used in combination with resveratrol against *B. cinerea* were reduced up to 16 times compared to the MICs of pyrimethanil used alone, indicating the strong synergistic antifungal activities between resveratrol and pyrimethanil.

We further examined the synergistic effects between resveratrol and pyrimethanil by the FICI values (see detailed description in Materials and Methods): the FICI for the moderately resistant isolates (GBW, TGM and GMR) and highly resistant isolates (BGM and BRB) ranged from 0.125–0.3125. However, the FICI of moderately resistant isolate SGB was higher than 0.5, indicating that the two-drug combination showed no synergistic antifungal activity against isolate SGB.

### 2.3. Synergistic Effect of Resveratrol and Pyrimethanil on Mycelial Growth

**Figure 1:**
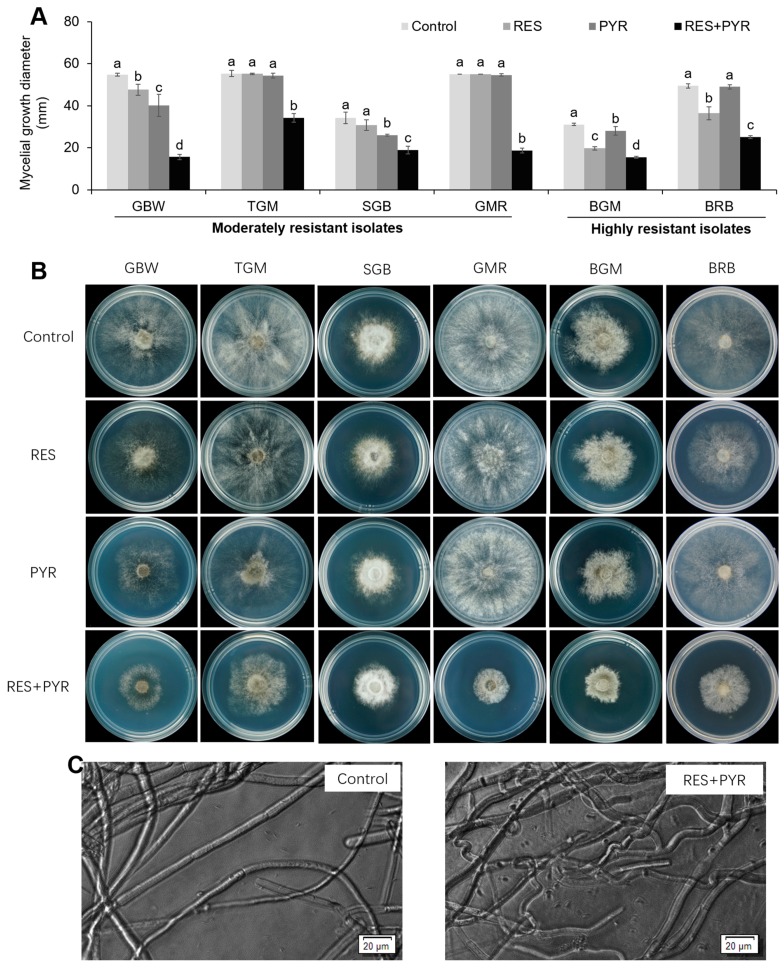
Inhibition on *Botrytis cinerea* mycelial growth by resveratrol and pyrimethanil treatment, alone or in combination. Six tested isolates were cultured on the PDA medium without (control) or with drugs (resveratrol at 50 mg/L, pyrimethanil at 3.125 mg/L and their combination). Mycelial growth diameter was measured (**A**) and photographed (**B**) at 72 h post incubation. Also, the morphology of untreated mycelial (control) or mycelia treated with two-drug combination for 72 h were observed and imaged by optical microscopy (**C**). RES and PYR represent resveratrol and pyrimethanil used alone, respectively. RES+PYR represent resveratrol and pyrimethanil used in combination. Data was presented as mean ± SE (standard error), and same isolate followed by different letters indicate significant (*p* < 0.05) differences according to Duncan’s multiple range test.

Mycelial growth of six *B. cinerea* isolates, including four moderately resistant isolates (GBW, TGM, SGB and GMR) and two highly resistant isolates (BGM and BRB), were assessed with treatment of resveratrol (50 mg/L) and pyrimethanil (3.125 mg/L), alone and in combination. As shown in Figure 1A,B, all tested isolates displayed significant sensitivity towards the combination of resveratrol and pyrimethanil (*p* < 0.05), even though some isolates (TGM, GMR and BRB) were not sensitive to pyrimethanil treatment. Microscopic examination of control (untreated) and drug combination-treated mycelia showed that the untreated mycelia were regular and homogeneous in morphology, while the mycelia treated with drug combination displayed curve and malformed appearance (Figure 1C).

### 2.4. Synergistic Effect of Resveratrol and Pyrimethanil on Conidia Germination

**Figure 2:**
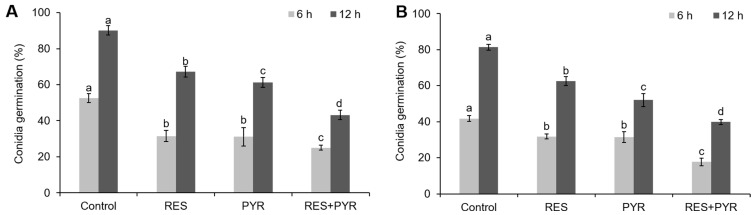
Suppression of *B. cinerea* conidia germination by resveratrol and pyrimethanil. Conidia suspension of *B. cinerea* isolate TGM (**A**) and BRB (**B**) was subject to different drug treatment and the conidia germination was measured at 6 h and 12 h post incubation. RES and PYR represent resveratrol and pyrimethanil used alone, respectively. RES+PYR represent resveratrol and pyrimethanil used in combination. Data was presented as mean ± SE (standard error), and same set of columns followed by different letters indicate significant (*p* < 0.05) differences according to Duncan’s multiple range test.

Next, we examined the suppression of *B. cinerea* conidia germination treated with two-drug, alone or in combination. Our result showed that individual treatment with each drug or treatment in combination significantly inhibit the conidia germination (*p* < 0.05), and the combination of resveratrol and pyrimethanil has strong synergistic antifungal activity against moderately resistant isolate TGM (Figure 2A) and highly resistant isolate BRB (Figure 2B). After 6 h of incubation, the conidia germination of moderately resistant isolate treated with resveratrol, pyrimethanil and combination group was lower than the control group by 0.7, 0.7 and 1.1-fold, respectively (Figure 2A). Consistent results were obtained from the highly resistant isolate under the same condition, as the conidia germination decreased to about 0.3, 0.3 and 1.4-fold compared to the control group, when treated with resveratrol, pyrimethanil and their combination, respectively (Figure 2B). The 12 h incubation yield consistent inhibitory activity as 6 h incubation. Overall, our results illustrated that two-drug combination treatment significantly suppressed *B. cinerea* conidia germination compared to untreated or single-drug treated group (*p* < 0.05) (Figure 2).

### 2.5. Synergistic Effect of Resveratrol and Pyrimethanil on Table Grape Gray Mold Disease Control

**Figure 3:**
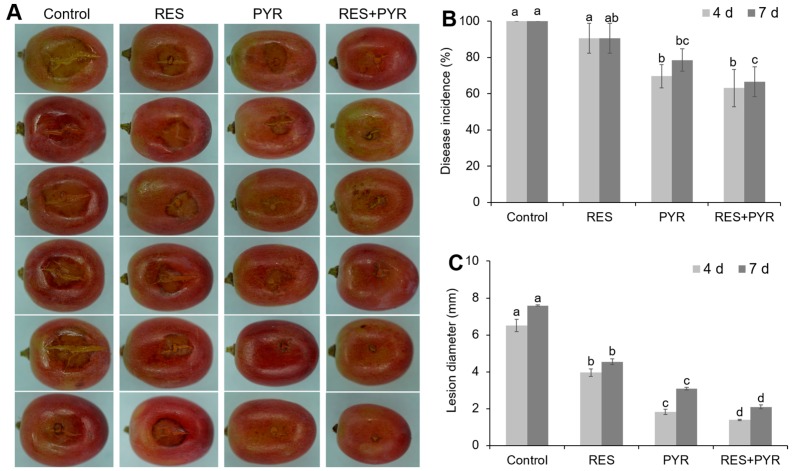
Control of table grape gray mold by resveratrol and pyrimethanil. Harvested grape berries were subject different drug treatment along with *B. cinerea* inoculation and kept at 22 °C. Symptoms were assessed and photographed at 7 days post inoculation (**A**). Disease incidence (**B**) and lesion diameter (**C**) were measured at 4 days and 7 days post inoculation, presented as bar charts. RES and PYR represent resveratrol and pyrimethanil treatment individually. RES+PYR represent resveratrol and pyrimethanil used in combination. Data was presented as mean± SE (standard error), and same set of columns followed by different letters indicate significant (*p* < 0.05) differences according to Duncan’s multiple range test.

In vivo effect of resveratrol (1 g/L), pyrimethanil (50 mg/L), and their combination on the control of gray mold on table grape were determined, and the disease symptom was documented in each treatment (Figure 3A). After 7 days storage at 22 °C, grape berries in two-drug combination treatment group displayed minimal lesion around the inoculation site (wound), while berries in the control (untreated) group and single-drug treatment group had apparent rot beyond the wounded area (Figure 3A). Two-drug combination treatment significantly reduced the disease incidence and lesion diameter compared to the control group (*p* < 0.05) (Figure 3B,C). After 4 days and 7 days of inoculation, the lesion diameter of gray mold treated with combination group was significantly lower than control group by 4.7 and 3.6-fold, respectively (*p* < 0.05) (Figure 3C). Furthermore, lesion diameter of berries treated with combination group significantly reduced compared to that in resveratrol- or pyrimethanil- treated group alone (*p* < 0.05) (Figure 3C), which reflected the synergistic effect between resveratrol and pyrimethanil on gray mold disease control in table grape.

## 3. Discussion

Resveratrol, a natural phenolic compound generated by various plants, including grapes, berries and peanuts was found to have antioxidant [32], anti-cancer [33], anti-inflammation [34], anti-aging [35] and anti-microbial effects against infectious disease of human [36,37]. In this study, resveratrol alone was proven to be effective in suppressing mycelial growth and conidia germination, which are consistent with previous studies in terms of the antifungal activity of resveratrol against *B. cinerea* [19,25,38].

The novel developed strategy that combines antifungal agents with natural compounds to manage various pathogenic fungal infections may be an ideal therapy, which effectively reduces toxicity and the emergence of multidrug resistance [39]. Resveratrol has been proposed as a promising strategy in combination with various antibiotics to cope with clinical resistant fungal infections and succeeded in reaching the excellent synergistic effects [40,41,42]. Our study represents the first report on resveratrol as a fungicide adjuvant which significantly enhanced the activity of pyrimethanil. The strong synergistic interaction between resveratrol and pyrimethanil against *B. cinerea* was confirmed by FICI method on six tested isolates. 

Conidium of *B. cinerea* shows remarkable flexibility in germinating in different environment once it adheres to the plant tissues. This is considered as the primary mean of diffusion and prevalence among plants by obtaining nutrients from the host plant and germinating into mycelium [2]. Our present finding showed that the combination of resveratrol and pyrimethanil significantly inhibited the conidia germination and mycelial growth, and this is of particular significance as it prevents the further dissemination of *B. cinerea*. The findings of this study clearly demonstrated that two-drug combination will help to suppress the development of pathogen, which consistent with previous study focused on the combination of plant extracts and fungicides to control several plant pathogens, including *Phytophthora infestans* [43], *Cercospora beticola* [44], *Uncinula necator* [45]. 

In addition, positive interaction of resveratrol and pyrimethanil on the control of gray mold was confirmed by inoculation assay with table grape cv “Crimson seedless”, further indicating the significant synergy between resveratrol and pyrimethanil in disease control. Although combined resveratrol and pyrimethanil treatment did not make significant difference on the disease incidence, it significantly reduced the diameter of disease lesion, compared to that in pyrimethanil treatment. Thus, the combination of resveratrol with pyrimethanil represents an attractive alternate approach for the new plant disease management strategy. Moreover, taken into consideration the price of resveratrol, grape pomace and pruning branch and leaves, which contained rich resveratrol and other phenolic compounds, can be considered as a good low cost source to obtain extract and applied together with pyrimethanil to control the gray mold.

## 4. Materials and Methods

### 4.1. Pathogen and Chemical Agents

**Figure 4:**
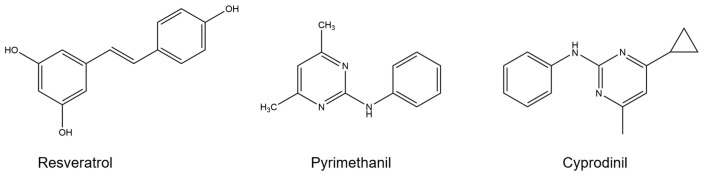
Chemical structures of resveratrol and two tested fungicides.

Six *B. cinerea* isolates (TGM, BGM, GMR, GBW, BRB and SGB) used in this study were isolated from infected berries and maintained on PDA (Potato dextrose agar medium, 200 g potato, 20 g dextrose and 15 g agar per liter) at 4 °C until use. Phenolic compound resveratrol (Figure 4) was purchased from Sigma-Aldrich (St. Louis, MO, USA, V900386) and with a purity grade higher than 98%. Anilinopyrimidine fungicides pyrimethanil and cyprodinil (Figure 4) used in this study were kindly provided by Department of Agriculture of Guangdong Province. Pyrimethanil was reagent grade and the stock solution prepared by dissolving into methanol, cyprodinil was the commercial product with the reagent grade of 50% (Syngenta, Suzhou, China, PD20142387) and was dissolved into sterile water.

Fresh table grape berries (cv Crimson seedless) with pedicels intact were carefully removed from the rachis by hand and surface sterilized in a 2% sodium hypochlorite solution for 2 min, washed with sterilized water and air dried at room temperature. 

### 4.2. Determination of the Fungicide Sensitivity of Tested Isolates 

To assess sensitivity of tested isolates to pyrimethanil and cyprodinil, mycelial plugs (5 mm diameter) were cut from the growing edge of a 3-day-old colony and transferred to PDA mediums containing different concentrations of pyrimethanil or cyprodinil, with methanol (0.8%, *v*/*v*) as solvent control. All plates were cultured at 22 °C in the dark for 72 h before measurement of colony diameter. Mycelial growth inhibition was presented as percentage as described by Sun et al. [46]. The effective concentration that reduced mycelial growth by 50% (EC_50_) for each isolate were calculated by linear regression of relative percentage of growth inhibition against log transformed fungicide concentration. This experiment was conducted in twice with four replicates for each concentration.

### 4.3. Classification of Resistance Level

To determine the sensitivity to pyrimethanil, the EC_50_ value of each isolate was used as described [47], with minor modifications. In this study, the concentration (0.4555 mg/L) with 5 times of the reported baseline sensitivity (0.0911 mg/L) was used as the threshold value. Mildly resistant isolates were defined as those having EC_50_ range from five times to ten times of threshold value (4.5 mg/L ≥ EC_5_0 > 2.5mg/L), moderately resistant isolates were defined as those having EC_50_ range from ten times to hundred times of threshold value (45 mg/L ≥ EC50 > 4.5 mg/L) and highly resistant isolates were defined as those having EC_50_ hundred times greater than threshold value (EC_50_ > 45 mg/L). The sensitivity of *B. cinerea* to cyprodinil was classified based on the sensitivity level described by Fernández-Ortuño et al. [48]. 

### 4.4. Determination of Minimal Inhibitory Concentration (MIC)

The MIC values of resveratrol and pyrimethanil against six isolates were determined using the two-fold serial microdilution method. Briefly, tested resveratrol and pyrimethanil were dissolved in methanol and added into PDA medium, and the final concentration of resveratrol and pyrimethanil were 1.25–160 mg/L and 0.3125–40 mg/L, respectively. An equal volume of methanol was added into PDA medium as solvent control. Mycelial plugs of 5 mm diameter were placed in the center of PDA plate and all plates were incubated at 22 °C in the dark for 72 h before colony diameter was measured. Three replicates were performed for each concentration and isolate treatment, and this experiment was performed twice.

### 4.5. Determination of MICs in the Drug Combination

To evaluate the drug interactions in vitro, the concentration of resveratrol and pyrimethanil was prepared in six concentrations, namely 1 × MIC, 1/2 × MIC, 1/4 × MIC, 1/8 × MIC, 1/16 × MIC and 1/32 × MIC. Then the mycelial growth at different concentrations of drug combination or solvent control (methanol) was measured after 72 h post incubation at 22 °C. MIC end points were defined as the lowest concentration of drugs prevented visible mycelial growth compared to the drug-free control. Each concentration was conducted in triplicate.

### 4.6. Analysis of Drugs Interaction

The in vitro interaction between resveratrol and pyrimethanil was assessed according to Loewe additivity (LA) theory using fractional inhibitory concentration index (FICI) to interpret drug interactions [49,50]. The FICI was defined by the equation: FICI = FIC_A_ + FIC_B_ = C_A_/MIC_A_ + C_B_/MIC_B_, where MIC_A_ and MIC_B_ represent the MICs of resveratrol and pyrimethanil when tested alone, C_A_ and C_B_ represent the MICs of resveratrol and pyrimethanil in combination, respectively. Interactions were categorized as synergism (FICI ≤ 0.5), indifference (0.5 < FICI ≤ 4.0) and antagonism (FICI > 4.0).

### 4.7. Mycelial Growth Assay

The effect on *B. cinerea* mycelial growth treated by resveratrol and pyrimethanil alone or in combination was tested by inoculating *B. cinerea* on PDA medium without drug (0.8% methanol as solvent control) or with individual or combined drugs (resveratrol at 50 mg/L and pyrimethanil at 3.125 mg/L, based on the MIC of each drug for all tested isolates). All plates were cultured at 22 °C in the dark for 72 h before colony diameter was measured. This experiment was conducted in twice with three replicates in each concentration treatment.

### 4.8. Conidia Germination Assay

To investigate the germination of *B. cinerea* conidia treated by resveratrol and pyrimethanil alone or in combination, experiment was conducted according to the method described by Xu et al. [25]. Conidia suspension (5 × 10^4^ conidia per mL) was prepared by washing conidia with PDB (Potato dextrose broth medium, 100 g potato, 20 g dextrose per liter) from fungal cultures and then treated with resveratrol (10 mg/L)/pyrimethanil (0.625 mg/L) by adding two-drugs individually or in combination into conidia suspension. Equal volume of methanol was added into conidia suspension as the solvent control. All treatments were incubated at 22 °C for different time intervals (0, 6 and 12 h) before conidia germination was documented. This experiment was conducted twice with three replicates for each instance.

### 4.9. Pathogenicity Assay

The inhibition effect on gray mold was assessed with table grape cv “Crimson seedless” treated by resveratrol and pyrimethanil individually or in combination. Table grape berries were wounded with dissected needles and inoculated with 5 μL of drug solutions (50 mg/L pyrimethanil, 1 g/L resveratrol individually or their combination). After air-dried at room temperature for 30 min, each berry was inoculated with 5 μL of conidia suspension (5 × 10^4^ conidia per mL) of *B. cinerea* isolate TGM at the wounded site [25]. Control berries were treated with 5 μL of methanol and inoculated with pathogen as described above. This experiment was repeated twice with four replicates, each consisting of 60 berries per treatment. All berries were placed in plastic containers and kept at 22 °C in the dark for 4 to 7 days, disease incidence was calculated by counting the percentage of infected berries and rot lesion diameter was measured. 

### 4.10. Statistical Analysis

All data in this study were subjected to ANOVA using Duncan’s multiple range tests and significant difference was determined at 5% level (IBM SPSS Statistics 22, USA). 

## 5. Conclusions

Our previous study showed that resveratrol exhibited significant antifungal activity against *B. cinerea*, which proved its efficacy as potential antifungal agent against gray mold. Interesting resveratrol showed excellent synergistic interactions with established fungicide pyrimethanil against mycelial growth and conidia germination of *B. cinerea*, which suggest that the combination of resveratrol and pyrimethanil can be expected to act as alternate treatment on the suppression of *B. cinerea*. This study further demonstrated that resveratrol and pyrimethanil could be used in combination for disease control of table grape gray mold at post-harvest period, providing evidence for the potential of resveratrol used as an adjuvant against this fungus.

## Figures and Tables

**Table 1:** Sensitivity of *Botrytis cinerea* isolates to pyrimethanil and cyprodinil.

Isolates	Pyrimethanil		Cyprodinil	
	Phenotype	EC_50_ (mg/L)	Phenotype	EC_50_ (mg/L)
GBW	MR	33.8	R	66.5
TGM	MR	39.9	MR	10.4
SGB	MR	22.8	MR	15.1
GMR	MR	11.9	S	1.3
BGM	HR	70.7	R	22.1
BRB	HR	88.0	MR	11.1

S, MR, R and HR indicate sensitive, moderately resistant, resistant and highly resistant.

**Table 2:** Interactions of resveratrol with pyrimethanil against *Botrytis cinerea* isolates.

Isolates	MICs (mg/L)				FICs			
	Alone		In Combination					
	MIC_A_	MIC_B_	C_A_	C_B_	FIC_A_	FIC_B_	FICI	IN
GBW ^MR^	40	0.625	2.5	0.15625	0.0625	0.25	0.3125	SYN
TGM ^MR^	80	1.25	5	0.078125	0.0625	0.0625	0.125	SYN
SGB ^MR^	40	0.625	2.5	0.3125	0.0625	0.5	0.5625	IND
GMR ^MR^	20	1.25	1.25	0.078125	0.0625	0.0625	0.125	SYN
BGM ^HR^	5	2.5	1.25	0.15625	0.25	0.0625	0.3125	SYN
BRB ^HR^	20	2.5	1.25	0.3125	0.0625	0.125	0.1875	SYN

MR and HR indicate the *B. cinerea* isolates moderately resistant and highly resistant to pyrimethanil, respectively. MICs were read as the minimal concentrations that inhibit mycelial growth compared with control group. MIC_A_ and MIC_B_ represent the MICs of resveratrol and pyrimethanil used alone, C_A_ and C_B_ represent the MICs of resveratrol and pyrimethanil used in combination, respectively. FICs indicate the fractional inhibitory concentration, FIC_A_ and FIC_B_ represent the FICs of resveratrol and pyrimethanil, respectively. FICI was calculated using the fomula: FICI = FIC_A_ + FIC_B_ = C_A_/MIC_A_ + C_B_/MIC_B_, and used as a determinant for drug interaction, defined as: FICI ≤ 0.5 for synergism, FICI > 4.0 for antagonism and 0.5 < FICI ≤ 4.0 for no interaction. IN, interpretation; SYN, synergism; IND, independence.
